# Construction and validation of prognostic nomogram and clinical characteristics for ovarian endometrioid carcinoma: an SEER-based cohort study

**DOI:** 10.1007/s00432-023-05172-5

**Published:** 2023-07-29

**Authors:** Wanlu Ye, Qing Wang, Yanming Lu

**Affiliations:** Department of Obstetrics and Gynecology, Shengjing Hospital of China Medical University, Shenyang, 110003 Liaoning China

**Keywords:** Ovarian endometrioid carcinoma, SEER database, Nomogram, Prognosis, Treatment

## Abstract

**Background:**

Ovarian endometrioid carcinoma (OEC) is the second most commonly occurring ovarian epithelial malignancy, but the associated prognostic factors remain obscure. This study aimed to analyze independent prognostic factors for patients with OEC and to develop and validate a nomogram to predict the overall survival (OS) of these patients.

**Methods:**

Clinical information of patients with OEC (2000–2019) was obtained from the Surveillance, Epidemiology, and End Results (SEER) database. Univariate and multivariate Cox analyses were used to identify independent prognostic factors, and nomogram models were constructed using independent prognostic factors. Receiver operating characteristic (ROC) curve, calibration plots, and decision curve analysis (DCA) were used to verify the accuracy and validity of the nomogram. Kaplan–Meier curves were used to compare the differences in OS and cancer-specific survival (CSS) among subgroups.

**Results:**

A total of 4628 patients with OEC were included, being divided into training (*n* = 3238) and validation (*n* = 1390) sets (7:3 ratio). On multivariate Cox analysis, AJCC stage, age, tumor size, differentiation, chemotherapy, and lymph node resection were significant predictors of survival outcomes (*P* < 0.05). Resection of 1–3 lymph nodes in early-stage OEC patients did not significantly prolong OS (*P* > 0.05), but resection of ≥ 4 lymph nodes in early-stage improved OS and CSS (*P* < 0.05). The OS of early-stage patients was not related to whether or not they received chemotherapy (*P* > 0.05). Lymph node resection and chemotherapy significantly improved the prognosis of patients with advanced OEC (*P* < 0.05). The c-index of nomogram prediction model was 0.782. ROC with good discrimination, calibration plots with high consistency, and DCA with large net benefit rate result in large clinical value.

**Conclusion:**

AJCC stage, differentiation, tumor size, age, chemotherapy, and lymph node dissection were prognostic factors of OEC. The constructed nomogram prediction model can effectively predict the prognosis of OEC patients and improve the accuracy of clinical decision-making.

## Introduction

Ovarian endometrioid carcinoma (OEC) originates from the ovary and its pathological histology is similar to that of primary endometrioid carcinoma. OEC was officially named by the International Federation of Gynecology and Obstetrics (FIGO) in 1964 (Long and Taylor 1964). OEC accounts for 10% of all ovarian epithelial malignancies and ranks only second to high-grade serous ovarian cancer (HGSOC) (Torre et al. 2018). OEC is the second most common ovarian epithelial malignancy and is often associated with endometrial lesions (Leskela et al. 2020), including endometriosis (EMs) and endometrial carcinoma. OEC tends to occur at a younger age (Zhou et al. 2021). Several studies have shown a significant association between OEC and EMs (relative risk [RR] 1.759; 95% confidence interval [CI], 1.551–1.995) (Zafrakas et al. 2014; Kim et al. 2014). The number of pregnancies (RR 0.78; 95% CI 0.74–0.83), age at menopause, tubal ligation, and hormone replacement therapy during menopause (RR 1.48; 95% CI 1.13–1.94) were shown to be strongly associated with the development of OEC (Liu et al. 2019; Wentzensen et al. 2016). The treatment strategies and the therapeutic regimes for OEC and the prognosis depend on the stage of the disease.

The Surveillance, Epidemiology, and End Results (SEER) database (https://seer.cancer.gov.) is an authoritative repository of cancer-related clinical data in the USA. It captures data pertaining to demographic characteristics of cancer patients, the disease status, treatment modalities, and basic prognostic information. SEER database can provide large datasets allowing for rigorous clinical research. Nomogram as a clinical prediction model that can be used to predict the probability of endpoint events for individual patients. The variables included in the nomogram are mainly derived from multifactorial Cox regression models. In the contemporary literature, nomograms have been developed for many tumors and have been shown to be useful in clinical practice.

The objective of this study was to develop and validate a nomogram prediction model for OEC based on data from the SEER database. To the best of our knowledge, this is the first study to establish a nomogram prediction model for OEC.

## Materials and methods

### Inclusion criteria and data collection

**Fig. 1:**
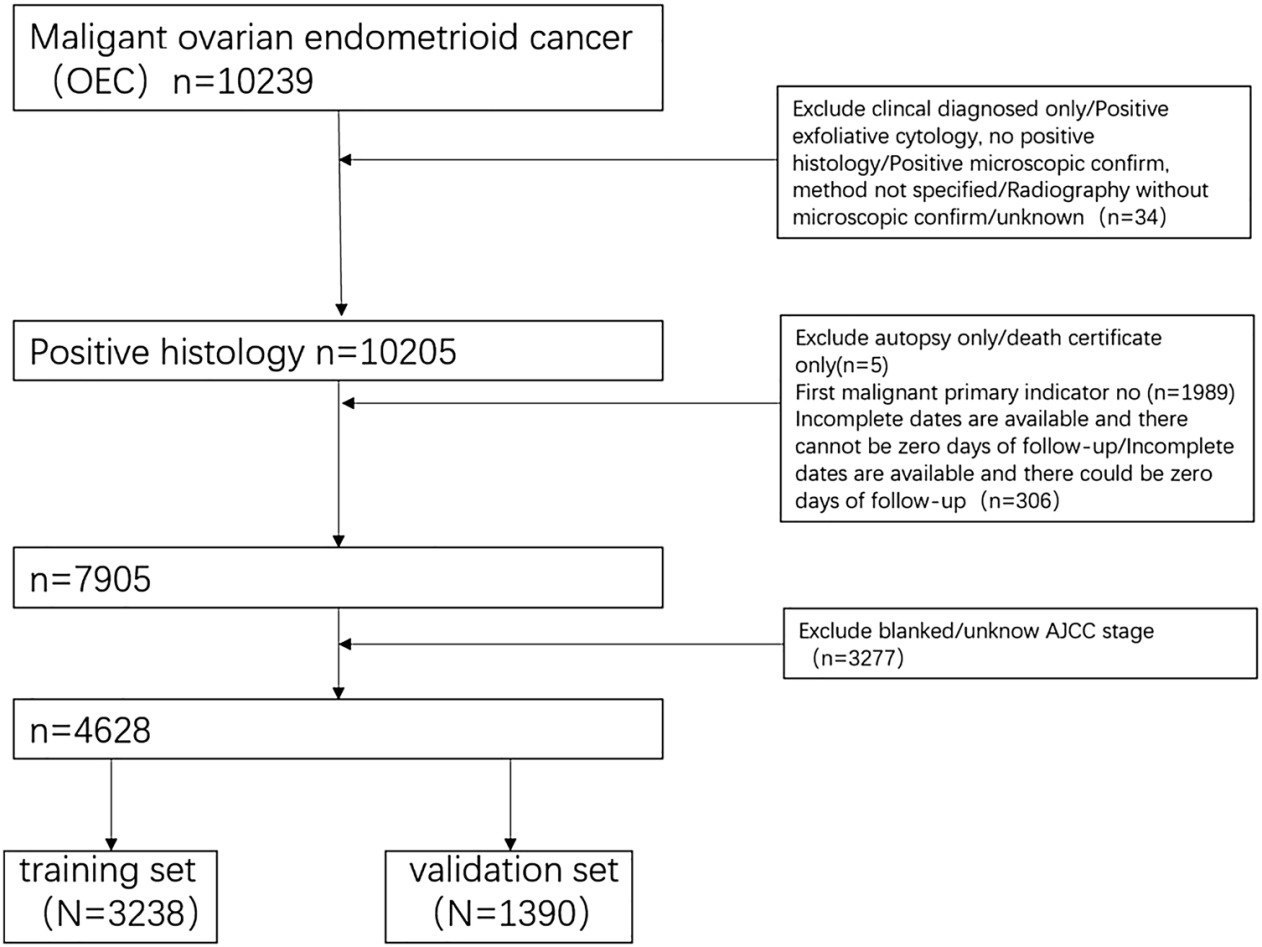
Flowchart of including and dividing patients

This retrospective cohort study utilized the SEER database of International Oncology Organization. The database contains information on patient's registration number, personal information, primary lesion site, tumor size, tumor code, treatment regime, and the cause of death. We selected all patients with ICD-O-3 Hist/behav, malignant codes 8380/3, 8381/3, 8382/3, 8383/3 with ovarian endometrioid carcinoma from the SEER database. The exclusion criteria were: patients that were not the first malignant primary indicator; lack of histological diagnosis; diagnosis based on autopsy or death records; and cases with incomplete data. Data pertaining to the following variables were retrieved: age, race, AJCC stage, degree of differentiation, tumor size, number of lymph nodes removed, and whether chemotherapy was administered. The main end points of this study were the 3-year, 5-year, and 10-year overall survival. SEER*Stat 8.4.0.1 was used to screen and collect data. The screening process is illustrated in Fig. 1. SEER belongs to a public database which does not require ethical approval.

### Clinical and demographic characteristics

Demographic characteristics for this analysis primarily included patient age (≤ 61, 62–73, and ≥ 74 years), race (American Indian, Asian, White, Black), AJCC stage (stage I, II, III, IV), grade (well differentiated, moderately differentiated, poorly differentiated, undifferentiated, unknown), tumor size (≤ 6.1 cm, > 6.2 cm), chemotherapy (yes, no), number of lymph nodes removed (none, 1–3, ≥ 4, unknown), and survival status (mainly including cancer-specific survival [CSS] and overall survival [OS]). Quantitative information such as age and tumor size was determined using X-tile software to determine the truncation values.

### Statistical analysis

Population data were presented in quantitative and percentage form. The study population was divided into a training set (*n* = 3238) and a validation set (*n* = 1390) in a ratio of 7:3. Differences in the baseline characteristics between the two groups were assessed using Chi-squared test. Clinical characteristics of patients with early-stage OEC and late-stage OEC were compared using Chi-squared test and Fisher test. Univariate and multivariate Cox regression analyses were performed to identify independent predictors of OS and CSS in OEC patients, and the results were presented as hazard ratio (HR) and 95% confidence interval (CI). OS and CSS curves were plotted using the Kaplan–Meier method. The predictor variables that were significant in the multivariate Cox analysis were used to build the nomogram. Nomogram plots predicting the OS of OEC patients at 3, 5, and 10 years were created, and calibrated in the training and validation sets, specifically using receiver operating characteristic (ROC) curve plots, C-index, calibration plots, and Decision curve analysis (DCA). All data and plots were produced using R version 4.2.0, SPSS27 software, and Graphpad prism 9. *P* values < 0.05 were considered indicative of statistical significance.

## Results

### Patient clinical characteristics

A total of 4628 patients (predominantly white; mean age: 56.2 years) in the SEER database were included. Most of the patients were at stage I (59%), and there were 763 (16.4%) in stage II, 836 (18.2%) in stage III, and 298 (6.4%) in stage IV. More than half of the patients received chemotherapy (60.6%) especially the patients at stage II-IV (77.6%). The number of patients who underwent lymph node dissection was 3178 (68.7%) and those who did not was 1337 (28.9%), the rest of were unknown. We divided the study population into a training set (*n* = 3238) and a validation set (*n* = 1390). There were no significant differences between the two groups with respect to baseline characteristics. The main clinical characteristics of the study population are presented in Table 1.

**Table 1:** Patient characteristics of ovarian endometrioid carcinoma

Subject	*N* = 4628, n (%)	Training set *n* (%) *N*1 = 3238	Validation set *n* (%) *N*2 = 1390	*P*
Age (years)				0.649
Mean	56.21	55.99	56.71	0.943
≤ 61	3123 (67.5)	2198 (67.9)	925 (66.5)	
62–73	972 (21.0)	674 (20.8)	298 (21.4)	
≥ 74	533 (11.5)	366 (11.3)	167 (12.0)	
Race				0.055
American Indian/Alaska Native	31 (0.7)	15 (0.5)	16 (1.2)	
Asian or Pacific Islander	508 (11.0)	357 (11.0)	151 (10.9)	
White	3837 (82.9)	2679 (82.7)	1158 (83.3)	
Black	232 (5.0)	173 (5.3)	59 (4.2)	
Unknown	20 (0.4)	14 (0.4)	6 (0.4)	
AJCC stage				0.311
I	2731 (59.0)	1887 (58.3)	844 (60.7)	
IA	1524 (32.9)	1073 (33.1)	451 (32.4)	
IB	148 (3.2)	104 (3.2)	44 (3.2)	
IC	1005 (21.7)	677 (20.9)	328 (23.6)	
INOS	54 (1.2)	33 (1.0)	21 (1.5)	
II	763 (16.4)	554 (17.1)	209 (15.0)	
IIA	200 (4.3)	146 (4.5)	54 (3.9)	
IIB	326 (7.0)	237 (7.3)	89 (6.4)	
IIC	204 (4.4)	141 (4.4)	63 (4.5)	
IINOS	33 (0.7)	30 (0.9)	3 (0.2)	
III	836 (18.2)	588 (18.2)	248 (17.8)	
IIIA	82 (1.8)	54 (1.7)	28 (2.0)	
IIIB	86 (1.9)	60 (1.9)	26 (1.9)	
IIIC	558 (12.1)	399 (12.3)	159 (11.4)	
IIINOS	110 (2.4)	75 (2.3)	35 (2.5)	
IV	298 (6.4)	209 (6.5)	89 (6.4)	
Grade				0.187
Well differentiated	1291 (27.9)	905 (27.9)	386 (27.8)	
Moderately differentiated	1565 (33.8)	1065 (32.9)	500 (36.0)	
Poorly differentiated	1080 (23.3)	777 (24.0)	303 (21.8)	
Undifferentiated	174 (3.8)	118 (3.6)	56 (4.0)	
Unknown	518 (11.2)	373 (11.5)	145 (10.4)	
Chemotherapy				0.72
No	1823 (39.4)	1270 (39.2)	553 (39.8)	
Yes	2805 (60.6)	1968 (60.8)	837 (60.2)	
Tumor size				0.995
≤ 6.1 cm	913 (19.7)	638 (19.7)	275 (19.8)	
> 6.1 cm	2925 (63.2)	2048 (63.2)	877 (63.1)	
Unknown	790 (17.1)	552 (17.0)	238 (17.1)	
Lymph nodes removed				0.34
None	1337 (28.9)	944 (29.2)	393 (28.3)	
1–3	466 (10.1)	337 (10.4)	129 (9.3)	
≥ 4	2712 (58.6)	1884 (58.2)	828 (59.6)	
Unknown	113 (2.4)	73 (2.3)	40 (2.9)	

### Survival analysis

**Fig. 2:**
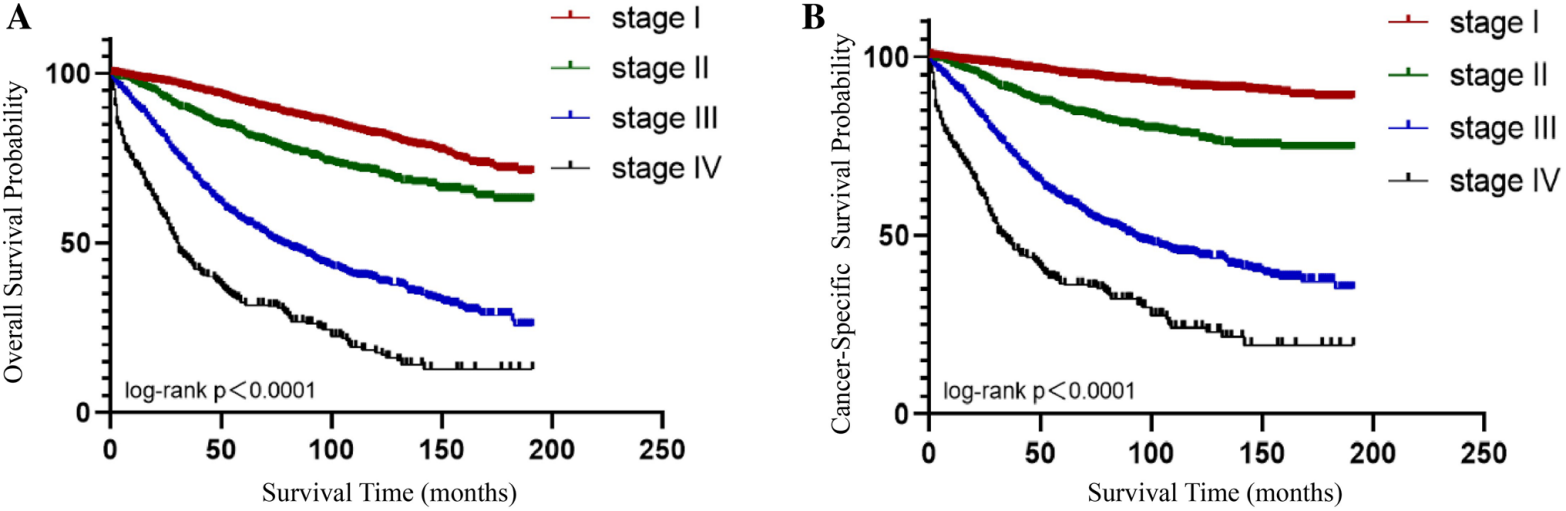
OS (**A**) and CSS (**B**) of the OEC in different AJCC stage. Patients with OEC have a different prognosis depending on their stage, and the higher the stage, the worse the patient's prognosis and the lower the OS and CSS

The OS and CSS curves for patients of different stages were performed using Kaplan–Meier survival analysis (Fig. 2). For early-stage patients, the 3-year overall survival rate was 93%, 5-year survival rate was 89%, and 10-year survival rate was 80%. For advanced-stage patients, the 3-year survival rate was 67%, 5-year survival rate was 59%, and 10-year survival rate was 46%. The 3-, 5-, and 10-year survival rates of patients in stage I were 93%, 89%, and 80%, respectively. The corresponding rates of patients in stage II were 84%, 79%, and 68%, respectively; those of patients in stage III were 63%, 51%, and 37%, respectively; and those of patients in stage IV were 37%, 30%, and 14%, respectively.

### Prognostic factors selection by univariate and multivariate Cox regression analysis

On univariate cox regression analysis, AJCC stage, age, tumor size, degree of differentiation, receiving chemotherapy, and lymph node dissection showed a significant effect on OS and CSS (*P* < 0.05). Race was not found to be a significant prognostic factor. On multivariate Cox analysis, all these factors were identified as significant predictors of the survival of OEC patients (*P* < 0.05) (Table 2).

**Table 2:** Univariate and Multivariate Cox analysis of prognosis factors of OEC

	Overall survival				Cancer-specific survival			
	Univariate HR (95%CI)	*P*	Multivariate HR (95%CI)	*P*	Univariate HR (95%CI)	*P*	Multivariate HR (95%CI)	*P*
Age (years)								
≤ 61	Ref		Ref		Ref		Ref	
62–73	1.698 (1.456–1.979)	< 0.001	1.432 (1.227–1.671)	< 0.001	1.405 (1.172–1.685)	< 0.001	1.112 (0.926–1.335)	0.255
≥ 74	4.257 (3.652–4.962)	< 0.001	3.271 (2.794–3.833)	< 0.001	2.661 (2.185–3.241)	< 0.001	1.920 (1.568–2.351)	< 0.001
Race								
American Indian/Alaska Native	Ref				Ref			
Asian or Pacific Islander	1.359 (0.430–4.296)	0.602			1.523 (0.373–6.223)	0.558		
White	1.752 (0.564–5.444)	0.332			1.850 (0.462–7.415)	0.385		
Black	2.407 (0.758–7.647)	0.136			2.601 (0.633–10.688)	0.185		
Unknown	0.000 (2.392E-67–2.529E + 58)	0.897			0.000 (0.000–3.450E + 70)	0.915		
AJCC								
Stage I	Ref		Ref		Ref		Ref	
Stage II	1.623 (1.338–1.970)	< 0.001	1.515 (1.239–1.851)	< 0.001	2.679 (2.097–3.423)	< 0.001	2.289 (1.778–2.947)	< 0.001
Stage III	4.597 (3.956–5.341)	< 0.001	3.528 (2.982–4.175)	< 0.001	8.518 (7.004–10.360)	< 0.001	5.857 (4.719–7.270)	< 0.001
Stage IV	9.643 (7.971–11.666）	< 0.001	7.139 (5.783–8.813)	< 0.001	17.272 (13.684–21.803)	< 0.001	10.972 (8.493–14.174)	< 0.001
Grade								
Well differentiated	Ref		Ref		Ref		Ref	
Moderately differentiated	1.783 (1.455–2.186)	< 0.001	1.4 (1.136–1.725)	0.002	2.558 (1.912–3.421)	< 0.001	1.800 (1.338–2.421)	< 0.001
Poorly differentiated	3.972 (3.270–4.824)	< 0.001	1.89 (1.529–2.337)	< 0.001	7.312 (5.559–9.618)	< 0.001	2.838 (2.121–3.799)	< 0.001
Undifferentiated	4.079 (3.017–5.514)	< 0.001	2.144 (1.569–2.931)	< 0.001	7.668 (5.286–11.124)	< 0.001	3.202 (2.178–4.707)	< 0.001
Unknown	2.741 (2.153–3.488)	< 0.001	1.823 (1.421–2.339)	< 0.001	4.535 (3.287–6.256)	< 0.001	2.635 (1.892–3.668)	< 0.001
Chemotherapy								
No	Ref		Ref		Ref		Ref	
Yes	1.261 (1.107–1.437)	< 0.001	0.782 (0.679–0.9)	< 0.001	1.784 (1.511–2.107)	< 0.001	0.876 (0.734–1.045)	0.141
Tumor size								
≤ 6.1 cm	Ref		Ref		Ref		Ref	
> 6.1 cm	1.426 (1.195–1.702)	< 0.001	1.298 (1.087–1.550)	0.004	1.473 (1.188–1.826)	< 0.001	1.257 (1.013–1.560)	0.038
Unknown	1.470 (1.188–1.818)	< 0.001	1.291 (1.043–1.599)	0.019	1.703 (1.323–2.192)	< 0.001	1.329 (1,031–1.713)	0.028
Lymph node removed								
None	Ref		Ref		Ref		Ref	
1–3	0.584 (0.473–0.722)	< 0.001	0.658 (0.532–0.814)	< 0.001	0.516 (0.398–0.669)	< 0.001	0.611 (0.471–0.794)	< 0.001
≥ 4	0.395 (0.346–0.451)	< 0.001	0.569 (0.496–0.654)	< 0.001	0.377 (0.322–0.442)	< 0.001	0.578 (0.491–0.680)	< 0.001
Unknown	0.449 (0.284–0.711)	< 0.001	0.548 (0.346–0.869)	0.011	0.398 (0.224–0.708)	0.002	0.476 (0.267–0.848)	0.012

### Treatment in early and advanced stage

**Fig. 3:**
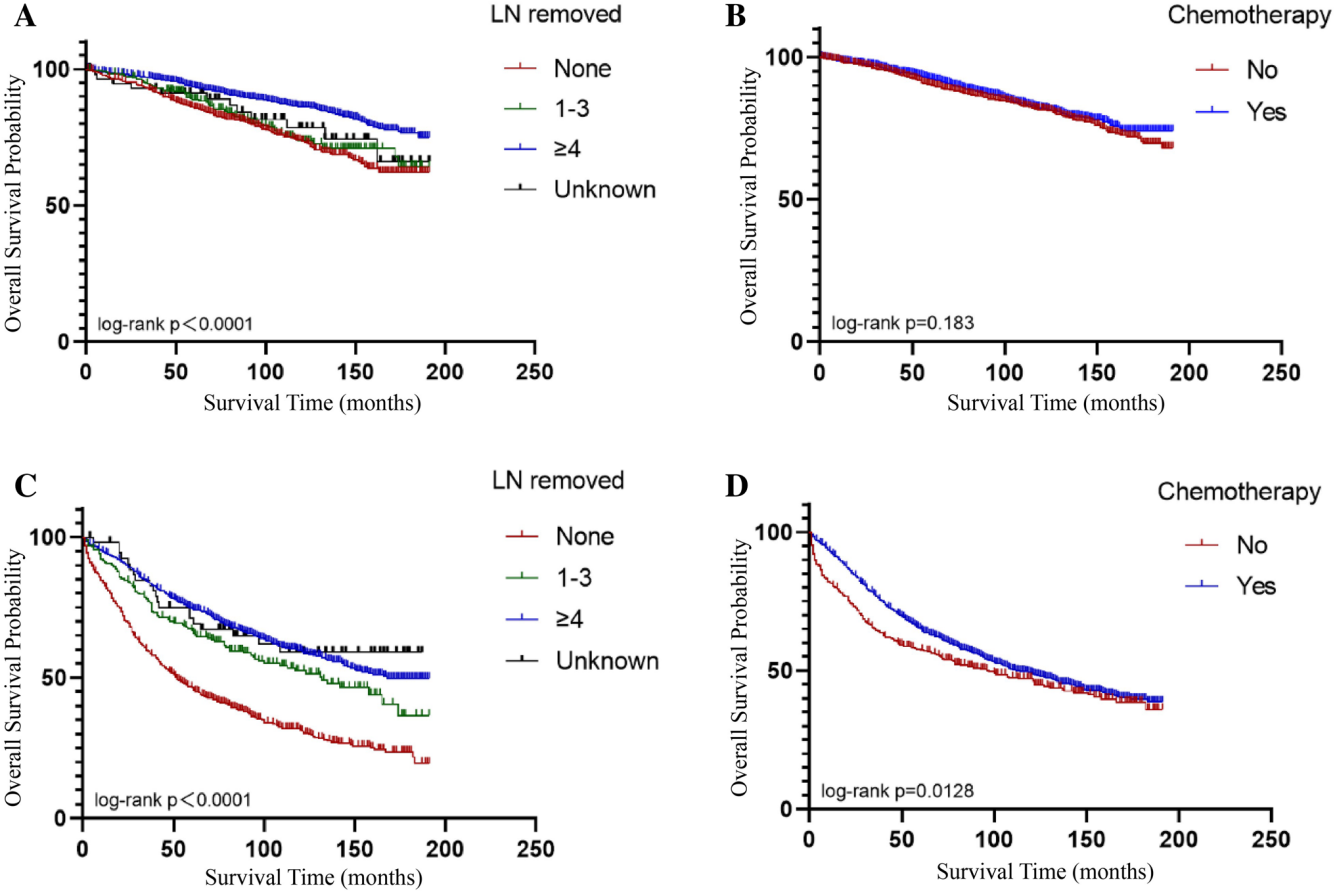
OS of the OEC within or without chemotherapy and lymph node removed in (**A**, **B**) early stage and (**C**, **D**) advanced stage. **A** Patients at early stage of OEC can significantly improve OS after undergoing resection of more than 4 lymph nodes, but resection of 1–3 does not improve prognosis. **B** Prognosis of patients with early OEC is independent of whether they receive chemotherapy or not. **C** Patients with moderate and advanced OEC can significantly improve OS after undergoing lymph node dissection. **D** Patients with moderate and advanced OEC can significantly improve OS after receiving chemotherapy

The results of univariate and multivariate Cox showed that the presence or absence of chemotherapy and the number of lymph nodes removed significantly improved the prognosis of OEC patients. For the purpose of analysis, patients in stage I were categorized as having early-stage disease and patients in stage II-IV were categorized as having advanced-stage disease. However, the use of different treatment modalities for patients with different stages of the disease may have biased the results. (Chemotherapy is less frequently used for early-stage patients compared to patients with advanced-stage disease.) Therefore, we opted for separate analysis of early and advanced-stage OEC patients (Table 3). The study showed that resection of 1–3 lymph nodes and whether or not chemotherapy was received had no significant effect on the OS of patients with early-stage OEC (*P* > 0.05), but resection of ≥ 4 lymph nodes improved the OS of patients with early-stage OEC (Fig. 3A) and CSS. Chemotherapy did not improve the OS of early-stage patients (Fig. 3B), but it prolonged the CSS. Lymph node dissection and chemotherapy significantly improved the prognosis of patients with advanced-stage disease (*P* < 0.05) (Fig. 3C/3D).

**Table 3:** Comparison between early-stage and advanced-stage OEC

Subject	Early stage (*n* = 2731)	Advanced stage (*n* = 1897)	*P*
Age (years)			< 0.001
Age-group (years)			
≤ 61	1941	1182	
62–73	537	435	
≥ 74	253	280	
Race			0.402
American Indian/Alaska Native	20	11	
Asian or Pacific Islander	288	220	
White	2275	1562	
Black	133	99	
unknown	15	5	
Grade			< 0.001
Well differentiated	1045	246	
Moderately differentiated	984	581	
Poorly differentiated	357	723	
Undifferentiated	59	115	
Unknown	286	232	
Chemotherapy			< 0.001
No	1399	424	
Yes	1332	1473	
Tumor size			< 0.001
≤ 6.1 cm	607	306	
> 6.1 cm	1696	1229	
Unknown	428	362	
Lymph nodes removed			< 0.001
None	640	697	
1–3	261	205	
≥ 4	1772	940	
Unknown	58	55

### Establishment and validation of nomogram prediction model

**Fig. 4:**
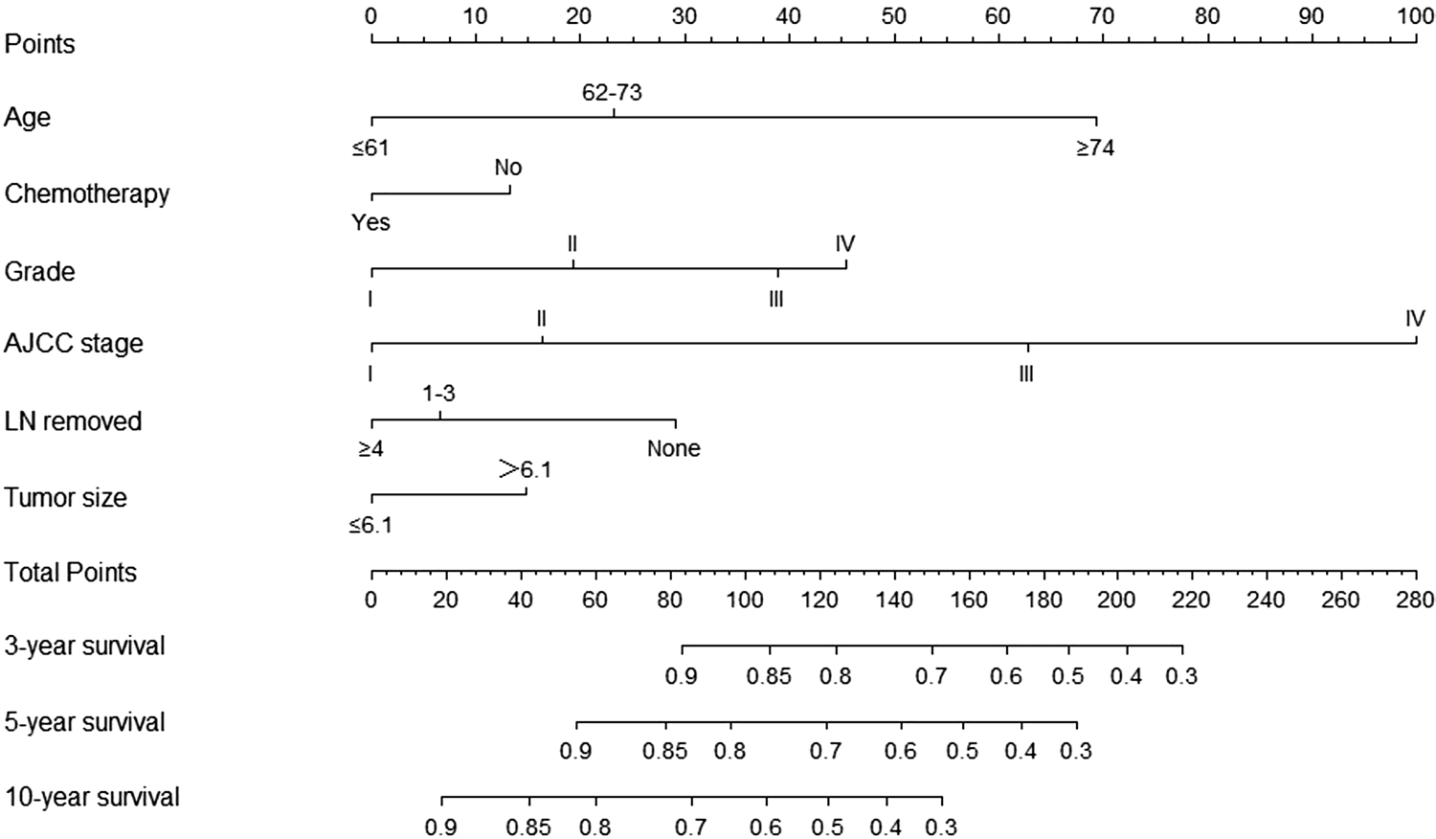
Nomogram predicting 3-, 5-, and 10-year OS for patients with OEC

**Fig. 5:**
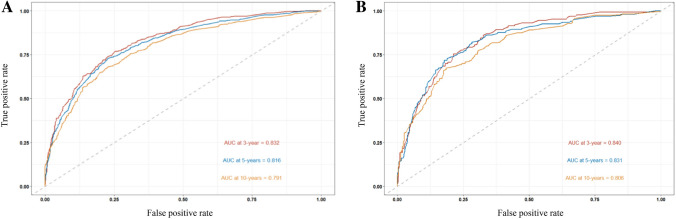
ROC of 3, 5, and 10 year of the training **A** and validation **B** sets

**Fig. 6:**
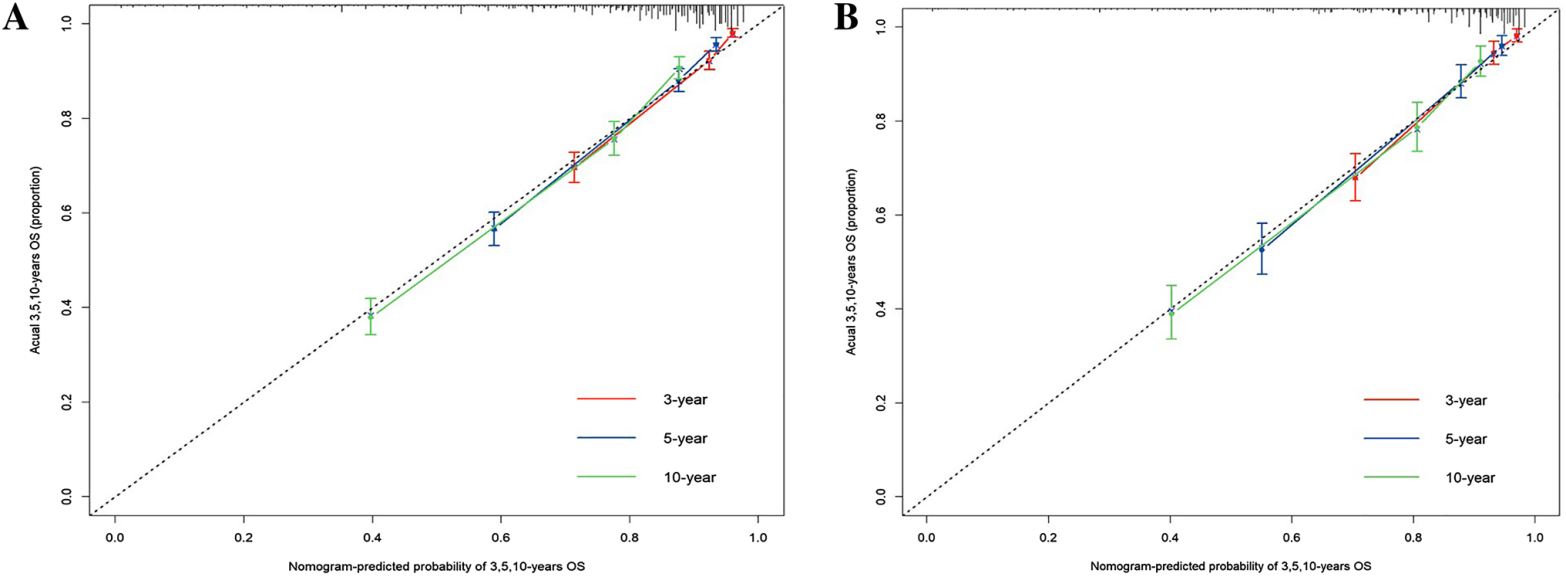
Calibration plots of the nomogram for predicting 3-, 5-, and 10-year OS. **A** Training group and **B** validation group

**Fig. 7:**
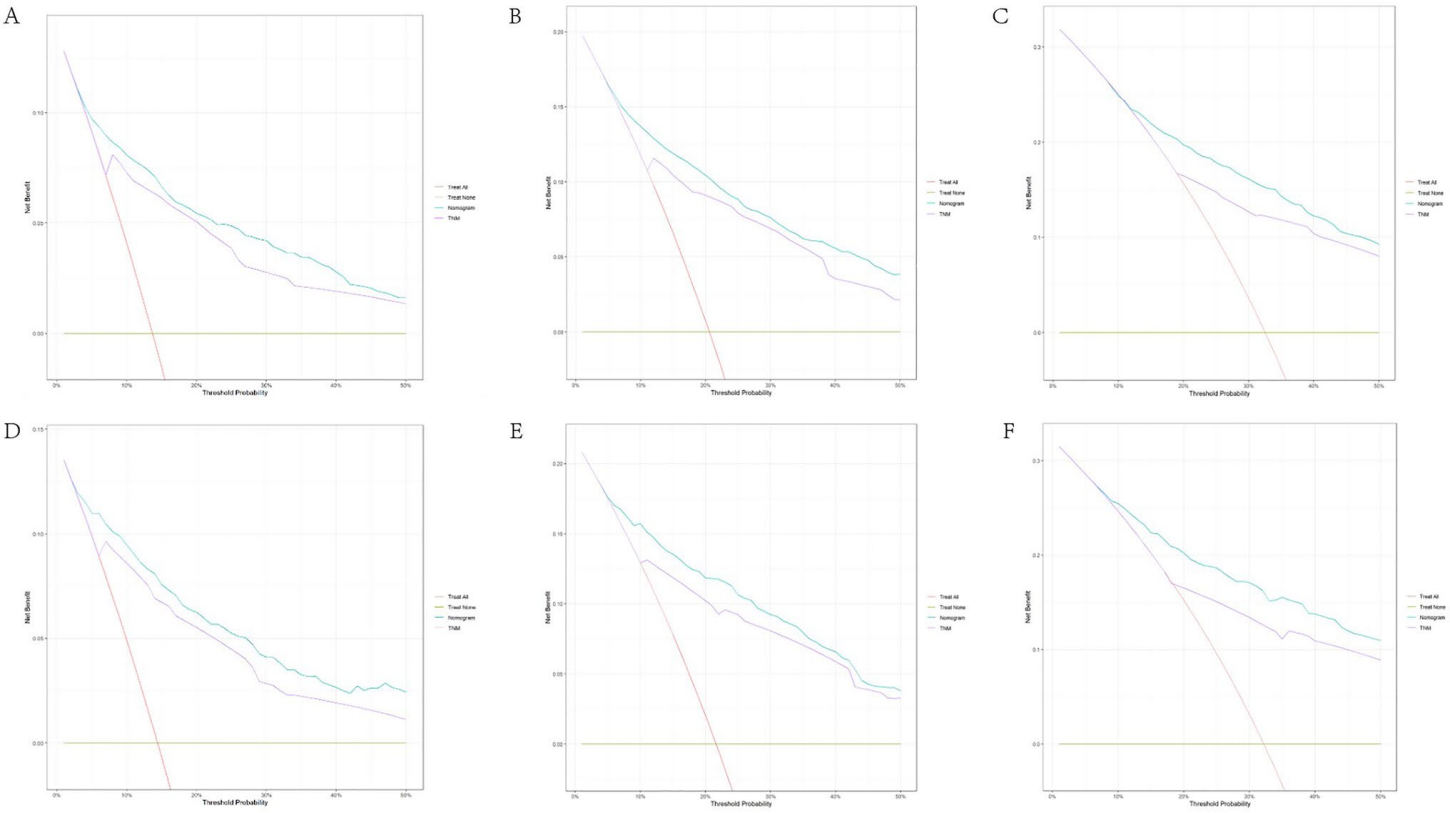
Decision curve analysis for evaluating the net benefit of nomogram and 6th AJCC TNM grading system. **A** 3-year net benefit in training cohort; **B** 5-year net benefit in training cohort; **C** 10-year net benefit in training cohort; **D** 3-year net benefit in validation cohort; **E** 5-year net benefit in validation cohort. **F** 10-year net benefit in validation cohort

Based on the results of multivariate Cox regression analysis, we developed a prediction model for OS by using the six prognostic factors of age, AJCC stage, grade, tumor size, whether to receive chemotherapy, and lymph node dissection (Fig. 4); the c-index of this model was 0.782. The “survivalROC” package in R was used to plot the ROC curve (Fig. 5). The area under the curve (AUC) of 3-, 5-, and 10-year survival in the training set was 0.832, 0.816, and 0.791, respectively; the AUC of 3-, 5-, and 10-year survival in the validation set was 0.840, 0.831, and 0.806, respectively. The calibration plots showed good agreement between nomogram prediction and observation at 3-, 5-, and 10-year in both training and validation groups (Fig. 6). The results indicated a high predictive accuracy of the model. The DCA of the training and validation sets showed that the 3-, 5-, and 10-year OS of the nomogram prediction model was higher than the DCA of TNM stage and other curves, obtaining a larger net benefit rate (Fig. 7).

## Discussion

Patients with early-stage OEC (stage I) accounted for the majority (59%) of the population screened from the SEER database. Diagnosis at an early stage implies a better prognosis for OEC; however, there may be inter-individual variability in this respect. Therefore, we downloaded and screened data from the SEER database and analyzed and constructed nomogram prediction models for OEC based on the significant prognostic factors. In this study, patient age, AJCC stage, tumor size, and treatment showed a significant impact on the prognosis of patients with OEC. Nomogram showed that older age, lower differentiation, higher stage, larger tumor, no chemotherapy, no lymph node dissection, or few lymph node dissections corresponded to higher scores and lower survival rates.

OEC is typically diagnosed at an early stage, and approximately 23% of patients with OEC have concomitant endometriosis (EMs) (Ju et al. 2019). Patients with OEC who also have EMs are mostly young and are typically diagnosed at an early stage due to obvious symptoms of EMs (Ju et al. 2019; Wang et al. 2013). This often translates to early treatment and better prognosis. The occurrence of OEC is significantly associated with EMs, and the prodromal lesions of OEC may originate from EMs in some cases. Therefore, the possibility of EMs combined with OEC needs to be taken into account. Although OEC typically affects younger women, it may also occur in elderly women, and our findings show that older patients have a poorer prognosis. Survival rates significantly differed between the three age-groups (≤ 61, 62–73, and ≥ 74 years) (*P* < 0.05), with worse prognosis in the older age-group. In a study based on the French ESME-Unicancer database, age was found to be one of the prognostic factors affecting OS in OEC patients, with lower OS in patients aged ≥ 50 years at diagnosis compared to those aged < 50 years (HR = 1.36, 95% CI 1.03–1.80, *P* < 0.05) (De Nonneville et al. 2021). This is likely attributable to the poorer physical condition of elderly patients and presence of comorbid cardiovascular, endocrine, and other diseases. The difficulty of chemotherapy and surgery in elderly patients due to physical factors may also contribute to the poorer prognosis.

Tumor lesions in patients with OEC are often unilateral (79–87%) (Moro et al. 2018; Lim et al. 2016), with a mean diameter of 11 cm. Our results show that tumor size is also an independent prognostic factor in OEC. The prognosis was significantly worse when the tumor diameter exceeded 6.1 cm, which was likely attributable to the fact that larger tumors imply faster tumor proliferation and possibly higher malignancy; larger tumors are often associated with the presence of subclinical lesions that are difficult to detect with the naked eye and difficult to remove surgically; finally, compressive symptoms caused by larger tumors tend to affect the quality of life of patients. Early OEC is often asymptomatic, but may present with irregular vaginal bleeding, pelvic masses, and abdominal distension. The common clinical manifestations of OEC are abdominal pain (19.9%), abdominal distention (19%), abdominal masses (8.1%), and vaginal bleeding (7.1%). Approximately 10–20% patients have concomitant endometrial cancer (Zaino et al. 1984). It is generally believed that OEC is mostly a primary disease, and when ovarian cancer and endometrial cancer are found at the same time, it is mostly considered as a simultaneous primary of both organs (Hascalik et al. 2005); in addition, it is also important to distinguish between primary and metastatic cancer, and the main points of differentiation are as follows (Moro et al. 2019): primary bilateral cancer: (1) no direct connection between the two tumors; (2) lesions are mainly located in the ovary and endometrium; (3) ovarian tumors are limited to the central part of the ovary, and endometrial cancer foci are less than 2 cm; (4) no myometrial infiltration or only slight myometrial infiltration; (5) no lymph and vascular infiltration; (6) endometrium with atypical hyperplasia; and (7) endometriotic foci in the ovary. OEC in combination with endometriosis often presents with abnormal vaginal bleeding. The presence of irregular vaginal bleeding suggests the possibility of concomitant endometrial disease, and further examination of the endometrium is recommended. Studies have shown that patients with stage I endometrioid endometrial cancer with synchronous stage I OEC are diagnosed earlier, have lower stage, and are less likely to receive radiotherapy. Moreover, the prognosis of patients with concomitant presence of both cancers was found to be similar to that of patients with endometrial cancer alone (*P* > 0.05) (Matsuo et al. 2017). In patients with combined endometrial cancer with OEC, cervical stromal invasion indicated a poorer prognosis (Yoneoka et al. 2019).

Tumor stage is an important prognostic factor, and TNM stage is a commonly used tool to evaluate patient prognosis. The 5-year survival rate of OEC patients typically exceeds 80% (Le Page et al. 2018; Krämer et al. 2020), and stage was shown to be an independent prognostic factor (Soovares et al. 2022). The 5-year survival rates of patients with stage I, II, III, and IV were reported to be 78%, 63%, 24%, and 6%, respectively (2020). In the present study, higher staging and lower differentiation corresponded to poorer prognosis, as well as higher scores in the nomogram prediction model, which are in agreement with previous studies.

The treatment strategy for OEC is comprehensive treatment with reference to serous ovarian cancer, which is mainly surgical. It mainly excludes occult metastases confined to the ovary. Studies have shown that comprehensive staging surgery in patients with stage I OEC is rarely seen for occult lesions that escalate the tumor stage (Padhy et al. 2019). The rate of lymph node metastasis in stage I OEC is 2.1% (Nasioudis et al. 2017), and our results show that for early-stage patients, the difference in the number of lymph nodes resected can improve the OS and CSS of patients; the survival difference was mainly attributed to the survival benefit conferred by resection of > 4 lymph nodes. However, the survival difference between patients with 1–3 lymph nodes resected and those without lymph node resection was not significant. In a previous study, lymph node dissection was found to be an independent predictor of DFS in patients with stage I OEC (*P* = 0.0276) (Zhao et al. 2017). A retrospective analysis showed that patients with stage I, low-grade OEC had a good prognosis and that adjuvant chemotherapy and staged lymph node dissection did not improve survival rates (Swift et al. 2022). In another study, patients who underwent pelvic and para-aortic lymph node dissection showed no significant improvement in OS (Yoshihara et al. 2021), and the difference in our results may be due to stratification of the number of lymph nodes dissected. Chemotherapy is a routine postoperative adjuvant treatment for ovarian cancer, but it is different for early-stage OEC. The results of this study showed that the prognosis of early-stage patients was not significantly affected by chemotherapy. The NCCN guidelines (Morgan et al. 2016) also recommend that patients with low-grade OEC (stage IA/IB) do not require adjuvant chemotherapy and that such patients should ideally be followed up; patients with low-grade OEC in stage IC should be considered for chemotherapy (3–6 cycles) or observation, but the rest of patients should receive chemotherapy. It was shown that after undergoing comprehensive staging surgery, patients with stage IA moderate-/low-grade OEC did not show increased 5-year survival rate after receiving chemotherapy (HR 1.092; 95% CI 0.954–1.249; *P* = 0.201) (Li and Zhu 2020), and patients at early stage did not receive adjuvant chemotherapy after undergoing comprehensive staging surgery. For young OEC patients at stage I, fertility-sparing surgery (FSS), i.e., removal of the adjacent adnexa on the affected side or bilaterally (with preservation of the uterus) is feasible if such patients have desire to reproduce. Based on the fact that removal of 1–3 lymph nodes and chemotherapy in early-stage patients does not improve the prognosis, FSS alone seems to be a plausible strategy for young patients at stage I. However, because of the risk of concomitant presence of endometrial disease, preoperative evaluation of the endometrium is required for patients undergoing FSS. For patients with moderate and advanced OEC, our study showed that chemotherapy and lymph node dissection can significantly prolong OS and CSS. In patients with moderate and advanced disease who have lesions involving the pelvis and upper abdomen at the time of initial treatment, i.e., stage IIB or higher, primary tumor cytoreduction (PDS) is considered, which aims to achieve R0 resection. Residual foci are significantly associated with patient prognosis. NCCN guidelines recommend postoperative adjuvant chemotherapy (paclitaxel + carboplatin, 6 courses) for stage II-IV patients, and our findings are consistent with previous studies. Also, FSS is not recommended for advanced OEC due to the presence of focal metastatic infiltration in most patients with moderate and advanced OEC (Bentivegna et al. 2016). Although the SEER database does not include data on endocrine, targeted and immunotherapy, PARP inhibitors may be used as maintenance therapy in newly diagnosed patients with moderate-/low-grade OEC with moderate and advanced OEC after receiving surgery and achieving complete or partial palliation with platinum-based drugs in first-line therapy according to guidelines and literature. Advanced OEC patients with MSI-H/dMMR/tumor mutation burden (TMB) ≥ 10 mutations/Mb may be considered for immunotherapy (Assem et al. 2018).

Some limitations of our study should be considered. A major limitation of the SEER database is the lack of data on recurrence, metastasis, and progression of tumors. Therefore, we could not analyze the prognostic factors for patients with recurrence, metastasis, and progression of OEC. The treatment strategy for patients with recurrent OEC is mostly referred to HGSOC. Recurrent patients with platinum-sensitive can be considered to receive secondary tumor cytoreduction, PARP inhibitor therapy, and anti-estrogen therapy for low-grade patients. Chemotherapy and targeted therapies are preferred for platinum-resistant relapsed patients. The SEER database does not provide data on EMs, so as a member of endometriosis-associated ovarian cancer, we were unable to compare the survival outcomes of OEC with or without concomitant EMs. However, according to previous studies, patients with OEC with concomitant EMs are younger, diagnosed earlier, and therefore have a better prognosis. The SEER database only provides data on the presence or absence of chemotherapy, but not on the specific regimen and dosage of chemotherapy, and endocrine therapy for OEC is not included.

## Conclusion

The results of our study showed that AJCC stage, differentiation, tumor size, patient age, whether to receive chemotherapy and lymph node dissection were prognostic factors for OEC. According to stratified analysis, early-stage patients may not benefit from chemotherapy, but resection of ≥ 4 lymph nodes was associated with improved prognosis of these patients. In patients with advanced-stage disease, chemotherapy and lymph node dissection were associated with significantly improved prognosis. The constructed nomogram prediction model can effectively predict the prognosis of OEC patients and improve the accuracy of clinical decision-making. In the future, our constructed nomogram prediction model needs further patient data for external validation.

## Data Availability

Publicly available datasets were analyzed in this study. These data can be found here: https://seer.cancer.gov/data-software/.
